# Aftermath of Grade 3 Ureteral Injury from Passage of a Ureteral Access Sheath: Disaster or Deliverance?

**DOI:** 10.1089/cren.2016.0109

**Published:** 2016-10-01

**Authors:** Roshan M. Patel, Zhamshid Okhunov, Kamaljot Kaler, Ralph V. Clayman

**Affiliations:** Department of Urology, University of California, Irvine, Orange, California.

**Keywords:** ureteral access sheath, ureteral injury, ureteroscopy, ureteral stent, endopyelotomy stent, urolithiasis, ureteral stones

## Abstract

***Background:*** The ureteral access sheath (UAS) has revolutionized the management of urinary pathology in the upper tract by providing rapid repeatable access to the upper urinary tract. However, in many practices, it remains a controversial tool in endourology given concerns of possible ureteral injury and presumed long-term sequela from those injuries. This case suggests that these concerns may be more hypothetical than real.

***Case Presentation:*** A 32-year-old female with a history of recurrent nephrolithiasis presented with left-sided symptomatic renal colic. She was found to have bilateral nephrolithiasis plus a left 6 × 5 mm proximal ureteral stone with associated moderate hydroureteronephrosis. The patient failed a trial of passage and as such was taken to the operating room for an elective ureteroscopy (URS) during which she sustained a Grade 3 ureteral splitting injury, measuring ∼2–3 cm, to the distal ureter from passage of the 16F UAS. At the end of the procedure a 7/10F endopyelotomy stent was placed. On follow-up URS at 6 weeks, there was no visual evidence of ureteral injury. A Lasix renal scan obtained 8 weeks after stent removal showed no evidence of obstruction.

***Conclusion:*** High-grade ureteral injuries sustained from UAS passage are rare. However, when injuries of this nature occur, the concern over long-term damage to the ureter may well be overstated.

## Introduction and Background

The ureteral access sheath (UAS) was first used in performing ureteroscopy (URS) in 1974 by Takayasu and Aso. This guide tube allowed for deployment of the ureteroscope, which at that time was only capable of passive deflection. Owing to difficulties in placing these early renditions of the UAS and the evolution of smaller, actively deflecting URS, the UAS fell from favor. With significant design improvements made by Clayman and colleagues in the late 1990s, the UAS was reintroduced.^1^ The UAS was noted to facilitate complex surgical tasks in the proximal ureter and kidney by allowing the surgeon to pass the flexible ureteroscope into and out of the ureter without difficulty for upper tract biopsies as well as removal of stone fragments. Additional benefits of UAS included an improved flow of irrigation fluid, shortened case times, less wear and tear on the flexible ureteroscope, and intraprocedural lower intrarenal pressures.^2–4^

Placing a UAS can result in adverse changes to the urothelium and smooth muscle layers of the ureter.^5^ Concerns were raised that these changes, although shown to be transient, would lead to long-term damage; moreover, many felt that should there occur a full thickness splitting of the ureter, the result would be a ureteral stricture. Despite recent studies showing that the rate of ureteral stricture is equivalent in URS performed with and without an access sheath,^6^ the concern over UAS-associated stricture formation persists. This case illustrates that even a severe injury such as ureteral splitting caused by UAS placement can result in complete healing of the ureter after prolonged stent placement.

## Presentation of Case

**FIG. 1.**
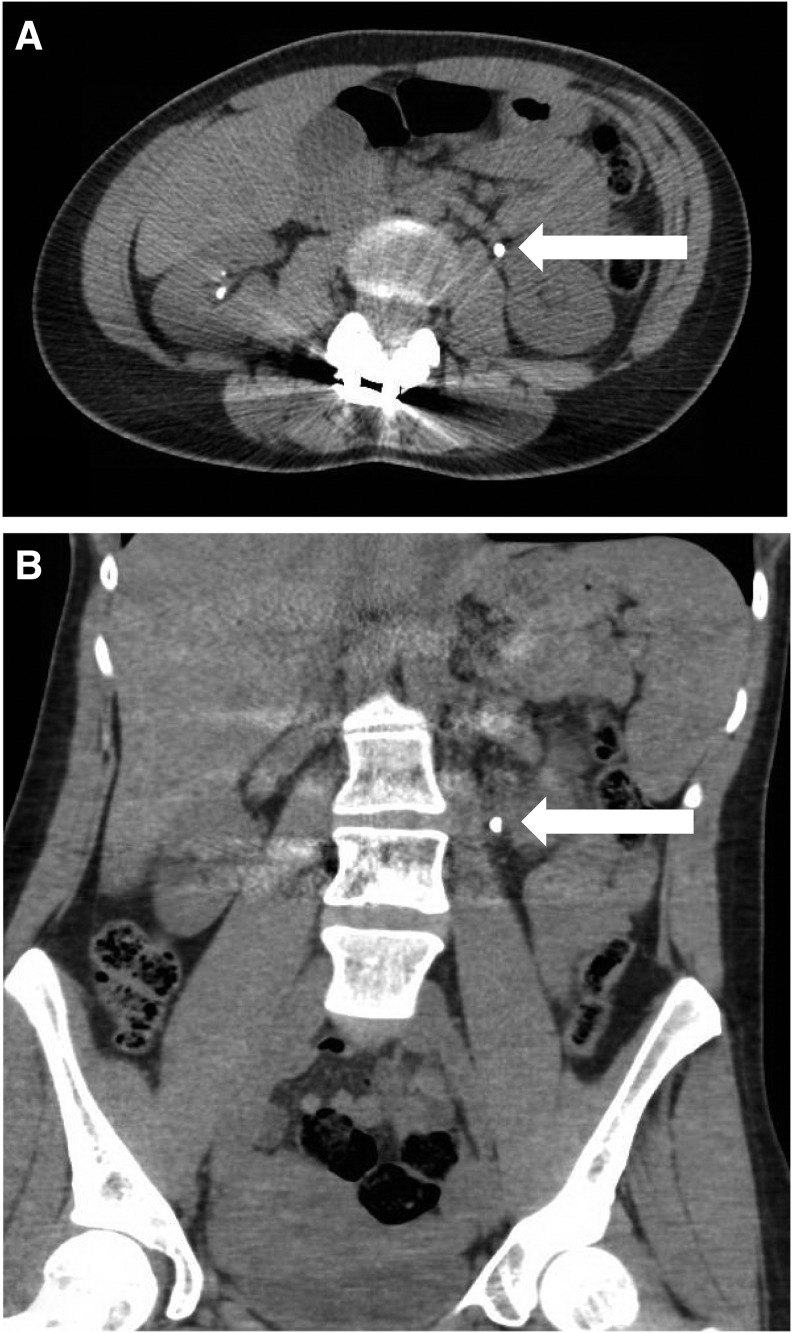
Representative selection of CT abdomen and pelvis images showing location of obstructing proximal ureteral stone, including axial **(A)** and coronal **(B)** cuts.

A 32-year-old female with a history of recurrent nephrolithiasis presented to an outside hospital emergency room with left flank pain, nausea, and vomiting. A noncontrast CT scan of the abdomen and pelvis revealed bilateral nephrolithiasis and a left 6 × 5 mm proximal ureteral calculus (Hounsfield units = 709) with associated moderate hydroureteronephrosis (Fig. 1). The patient was without signs of infection and was discharged to home; she returned to the emergency room twice more within the week and was instructed to take pain medications and continue taking tamsulosin 0.4 mg nightly. She presented to our office at day 9 with persistent renal colic and elected to proceed with URS. Of note, her urine culture was sterile and her blood chemistries and white blood cell count were unremarkable.

**FIG. 2.**
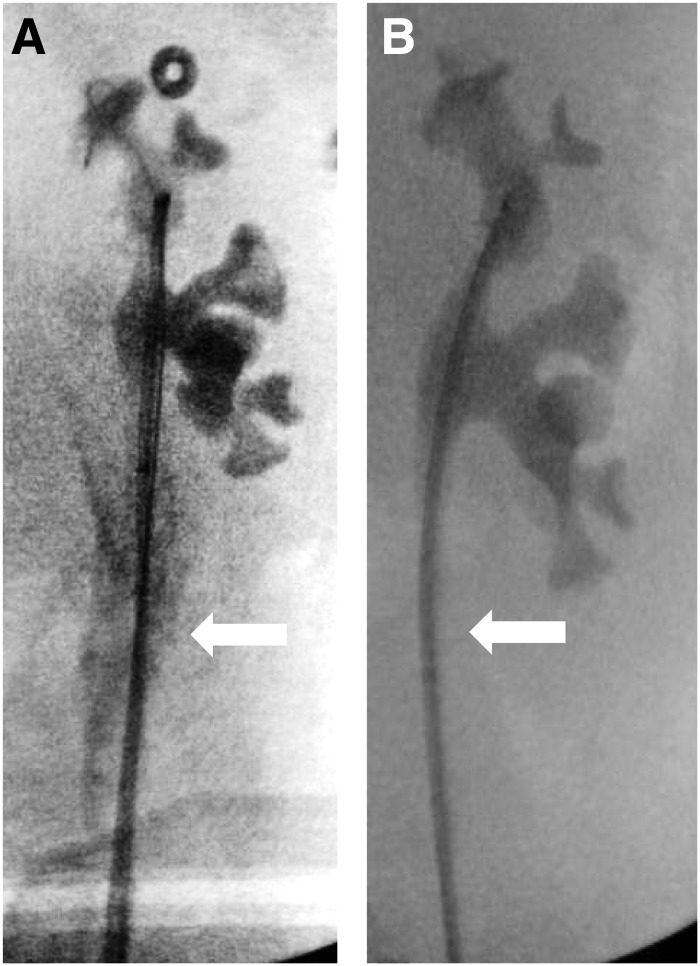
Representative selection of intraoperative fluoroscopic images showing contrast extravasation at initial ureteroscopy **(A)** and resolution of ureteral injury after stent placement 6 weeks postoperation **(B)**.

With the patient under general anesthesia, in a dorsal lithotomy position, flexible cystoscopy was performed. The left ureteral orifice was cannulated with a 0.035 inch Nitinol guidewire, which was advanced beyond the stone under fluoroscopic guidance. An 8F Amplatz catheter was then passed over the Nitinol guidewire and the Nitinol guidewire was replaced with a 0.035 inch Amplatz super-stiff guidewire. The 8F Amplatz catheter was removed. A 16F, 30 cm UAS was advanced to the mid ureter. A Karl Storz 7.5F flexible ureteroscope was inserted, and the stone was fragmented using a 365-micron holmium laser fiber at 2 J and 20 Hz; all visible fragments were basketed and removed. The ureter was inspected as the access sheath was removed. The proximal ureter revealed no abnormalities, other than some edema at the site of the stone bed; however, just distal to the iliac vessel crossing, there was a full thickness separation of the ureter out to periureteral fat (Supplementary Video S1). The injury measured ∼2–3 cm and was consistent with a Grade 3 ureteral injury as described by Traxer and Thomas.^5^ A retrograde pyelogram confirmed extravasation of contrast at the location of the ureteral injury (Fig. 2). As such, a 26 cm 7/10F endopyelotomy stent was left in place with the 10F portion traversing the lower ureter. A 16F Foley catheter was left in place for 3 days.

The patient had significant anxiety about cystoscopy with stent removal in the office and as such she was taken to the operating room 6 weeks after her initial URS. The stent was removed over a 0.035 inch Nitinol guidewire and the 8.5F Storz digital flexible ureteroscope was passed over the guidewire, which was then removed. Inspection of the kidney and ureter revealed no residual stones and a widely patent ureter with intact urothelium covering the site of the prior ureterotomy (Supplementary Video S1). A retrograde pyelogram showed no extravasation (Fig. 2). Eight weeks after stent removal, the patient had a Lasix renogram. The T½ on the affected left side was 2.83 minutes and the split function of the left kidney was 51.3%.

## Discussion and Literature Review

Significant advances in URS and more recently URS technology have facilitated the broad application of endoscopy to all aspects of upper tract pathology. In this regard, the UAS has been shown to reduce procedural time and reduce costs without any increase in complications.^7^ In addition, the UAS decreases renal pelvic pressure during URS thereby reducing the chances of pyelovenous backflow, and potential urosepsis.^2–4^ Although the data on stone-free rates utilizing a UAS are mixed, the UAS provides a tremendous advantage in which multiple reinsertions of the ureteroscope are required to basket stone fragments.

The investigation into the possible ischemic effects of the UAS has only been investigated in the pig.^8^ In this solitary animal study, ureteral blood flow was measured using laser Dopper flow measurements before and after UAS placement. A notable initial decrease in blood flow occurred when a 16F UAS was deployed; however, this initial decrease in blood flow reversed itself within 20 minutes. Furthermore, the authors reported no evidence of histologic ischemic damage 72 hours after UAS removal. Nonetheless, concerns about potential late strictures continued to be expressed although without substantiation.

In this case, a 16F UAS was initially passed as the patient had been on tamsulosin 0.4 mg nightly for 1 week before surgery; in our experience, pretreatment with an alpha-blocker for 1 week appears to relax the ureteral smooth muscle and facilitates passage of larger access sheaths. Irrigation fluid flow rate increases up to sevenfold with increasing sheath diameter,^9^ and the larger sheath facilitates passage of the larger digital or dual channel ureteroscopes while enabling the removal of stone fragments up to 4 mm in diameter.^10^

Indeed, the incidence of ureteral strictures among patients undergoing URS with and without a UAS is similar.^11^ The key to avoiding injury is to not exert any excessive force on the sheath during placement and thus place a UAS that easily slides up the ureter.^12^ Ironically, the dreaded complication of an iatrogenic ureterotomy that hypothetically might lead to a late ureteral stricture, indeed, has no different appearance than a well done endouretertomy performed to treat a short ureteral stricture after an errant URS performed without a UAS. This case demonstrates that even with a Grade 3 injury after UAS passage, the ureter can heal in a widely patent, completely functional manner.

## Conclusion

A consensus exists among many urologists that a UAS should be used on a highly selective rather than routine basis because of concerns of ureteral injury and stricture formation. However, URS is performed more cost efficiently with an access sheath and the complication rate of URS with an access sheath is no greater than without one. Indeed, as documented by this case, even a significant ureterotomy may heal without any sequela after long-term stent placement.

## Supplementary Material

Supplemental data

## Abbreviations Used

CT: computed tomography
URS: ureteroscopy
UAS: ureteral access sheath
